# Supplementary material for: An accurate wearable hydration sensor: Real-world evaluation of practical use
Source: PLoS One. 2022 Aug 24;17(8):e0272646. doi: 10.1371/journal.pone.0272646 (PMC9401113; doi:10.1371/journal.pone.0272646)
Supplement: S1 Table — Calculated metrics were as follows: Mean bias including mean bias %, MAPE (mean absolute percentage error) including MAPE standard deviation (MAPE SD) and 95% confidence interval (MAPE 95%CI), %NRMSE (percent normalized root mean square error), MAE (mean absolute error) including MAE standard deviation (MAE SD). Formula abbreviations are as follows: W = Smartwatch-DBM measurement; B = balance standard comparison measurement. (PDF) [file pone.0272646.s001.pdf]

**Metric**	**Formula**
Mean Bias	$\sum_{i=1}^{n}[(B-W)/n]$
Mean Bias %	$\left[(Mean\ Bias)/\left(\sum_{i=1}^{n} B/n\right)\right]\times\ 100$
MAPE	$\frac{100}{n}\sum_{i=1}^{n}\lvert(B-W)/B\rvert$
MAPE SD	$\sqrt{\frac{1}{n}\sum_{i=1}^{n}\left[\lvert B-W\rvert_i-\left(\frac{100}{n}\sum_{i=1}^{n}\lvert(B-W)/B\rvert\right)^2\right]}$
MAPE 95%CI	$MAPE \pm 1.96\times\frac{MAPE\ SD}{\sqrt{n}}$
%NRMSE	$100\times\frac{\sqrt{\frac{\sum_{i=1}^{n}(B-W)^2}{n}}}{\frac{\sum_{i=1}^{n}(B-W)^2}{n}}$
MAE	$\frac{\sum_{i=1}^{n}\lvert B-W\rvert}{n}$
MAE SD	$\sqrt{\frac{1}{n}\sum_{i=1}^{n}\lvert B-W\rvert_i-\left(\frac{\sum_{i=1}^{n}\lvert B-W\rvert}{n}\right)^2}$
