# Supplementary material for: An accurate wearable hydration sensor: Real-world evaluation of practical use
Source: PLoS One. 2022 Aug 24;17(8):e0272646. doi: 10.1371/journal.pone.0272646 (PMC9401113; doi:10.1371/journal.pone.0272646)
Supplement: S1 File — (PDF) [file pone.0272646.s003.pdf]

03/27/17

Spectrophon Dehydration Body Monitor Accuracy Study

NCT03229109

Authors:

Gleb Zilberstein, Anatoly Kreinin,

Dmitry Rodin, Albert Pinhasov

1. *Kiryat Carmel Hospital, Israel*
2. *Ariel University, Israel*
3. *Spectrophon LTD, Rehovot, Israel*
4. *Maale HaCarmel Mental Health Center, Israel*

Protocol date: 03/27/2017, ver.: 3

-2017-

1. Study protocol

Healthy adults (n=200) in different age and gender groups were recruited for the study. Samsung Gear S2 smartwatch with Spectrophon DBM attached to the bottom of the smartwatch were placed on the right wrist of each participant. At the same time, Samsung Gear Fit2 sport band with Spectrophon DBM attached to the bottom of the band was placed on the left wrist of the participant. Each participant was subjected to moderate physical activity. Data from Samsung Gear S2 and Samsung Gear Fit2 was obtained simultaneously. In parallel, subject weight was also monitored using commercially available digital balancers (Shekel B-200-P).

The evaluation of the measurement accuracy of the Spectrophon DBM was defined as the difference between subject weight change during the exertion (due to sweating and water consumption) and the volume of sweat detected by Spectrophon DBM.

All experiments were conducted indoors under ambient temperature (18 ^{o}C) and humidity (40-60%) .

1.1. Experimental groups:

Number of subjects: 200 Age range: 18-50+

18-25 years: 50 participants 26-35 years: 50 participants 36-45 years: 50 participants 46-50 years: 50 participants **1.2. Inclusion criteria:**

1. Age: older than 18, both gender.
2. Ability and willingness to sign an informed consent form for participation in the study.

Exclusion criteria:

1. Presence of cardiologic or vascular disease.
2. Evidence of any other serious medical disorder.
3. Pregnancy

2. Procedures:

Participants were weighed prior to the experiment (no clothing after maximal drying) and then subjected to physical activity (walking on the treadmill).

2.1. Activity protocol:

T0: Initiate exercise

T1: T0+15 min - Stop exercise, Rest T2: T0+25 min - Initiate exercise

T3: T0+40 min – Stop exercise, Rest T4: T0+50 min - Initiate exercise T5: T0+65 min – Stop exercise

T6: T0+75 min - Initiate exercise T7: T0+90 min – Stop exercise

Total duration of study: 90min.

2.2 Intensity of exercises:

Participants could choose high or low intensity of exertion: **a. High**: 0:00-0:01 – preparation;
0:01-0:05 – 5.5km/h;
0:05-0:10 – 6.0km/h;
0:10-0:15 – 6.5km/h;

b. Low: 0:00-0:01 – preparation; 0:01-0:05 – 5.0km/h;
0:05-0:10 – 5.5km/h;
0:10-0:15 – 6.0km/h;

2.3 Data recording:

After DBM application was activated, DBM started recording data (sweat rate and total salt in sweat) every 20 sec. and automatically saved results into archive on a mobile phone linked to Samsung Gear S2 or Samsung Gear Fit2 by Bluetooth. Manual recording of data was conducted during breaks (between phases T1-T2, T3-T4, T5-T6 and after T7).

Participants were also weighed during each break (no clothing after maximal drying).

2.4. Drinking

During the procedure, subjects could drink up to 500 ml of water. The weight of the bottle was measured and recorded after drinking during breaks. The difference was subtracted to the weight loss calculation.

2.5 Restrictions:

In this experiment, we avoided:

1. Urination during test (empty before T0)
2. Weight loss should not exceed 2%

Participants could cancel the experiment at any point of the procedure if desired.

3. Statistical analysis

Data obtained from Spectrophone DBM will be compared to the subject's total weight loss for every participant. The measurement error distribution will be estimated.

Principal investigator

Prof. Anatoly Kreinin

Signature
