# Supplementary material for: An accurate wearable hydration sensor: Real-world evaluation of practical use
Source: PLoS One. 2022 Aug 24;17(8):e0272646. doi: 10.1371/journal.pone.0272646 (PMC9401113; doi:10.1371/journal.pone.0272646)
Supplement: S2 File — (PDF) [file pone.0272646.s004.pdf]

**שם הנוהל: נוהל לניסויים רפואיים בבני-אדם**	**תאריך: מאי 2014**
**טופס 7**	
**אישור מנהל המוסד הרפואי לביצוע ניסוי רפואי**	

תאריך: 8.5.17

לכבוד
פרופ' אנטולי קריינין
החוקר הראשי
<u>מנהל מחלקה 5א'</u>

פרופ' / ד"ר נכבד/ה,

הנדון: **<u>אישור לביצוע ניסוי רפואי בבני-אדם</u>**

בהתאם לבקשתך מיום: **27.03.17** ניתן בזה אישור לביצוע הניסוי הרפואי לפי מסמכי הבקשה

פרטי הניסוי

מספר בקשה בוועדה מוסדית: **04/17**	סוג הניסוי: **אמ"ר**.
מספר הבקשה במשרד הבריאות:	מספר רישום ב-NIH:
נושא הניסוי : **אימות פונקציונלי של חיישנים ביולוגיים עבור מעקב פיזיולוגי.**	
שם מוצר המחקר: **Spectrophon Dehydration Body Monitor based on Samsung PPG/HRM Gear 2S**	שם היצרן: **Spectrophon Ltd.**
ניסוי רב-מרכזי בארץ: **X לא**	

מסמכי הניסוי

פרוטוקול הניסוי- שם/מספר:	גרסה: **3**	תאריך: **27.03.17**
טופס הסכמה- שם/מספר:	גרסה: **3**	תאריך: **31.03.17**
חוברת לחוקר- שם/מספר:	גרסה: **2**	תאריך: **02.04.17**
מסמך איכות מוצר - שם/מספר:	גרסה:	תאריך:
טופס 11- גרסה: **1**	תאריך: **18.04.16**	

**שם הנוהל: נוהל לניסויים רפואיים בבני-אדם**	**תאריך: מאי 2014**
**טופס 7**	
**אישור מנהל המוסד הרפואי לביצוע ניסוי רפואי**	

בתוקף ההסמכה שקיבלתי מהמנהל הכללי של משרד הבריאות, לתת אישור כ"מנהל" לעשיית ניסוי רפואי בבני-אדם, במוסד הרפואי, לאחר שהבקשה אושרה על-ידי ועדת הלסינקי המוסדית בתאריך: **03.05.17**, ולאחר ששוכנעתי כי הניסוי הרפואי הנו בהתאם לעקרונות של הצהרת הלסינקי ותקנות בריאות העם (ניסויים רפואיים בבני-אדם) תשמ"א-1980, וכי חוזה ההתקשרות בין היזם, החוקר הראשי והמוסד הרפואי עומד בדרישות הנוהל לניסויים רפואיים בבני אדם, הנני מאשר את ביצוע הניסוי בכפוף לתנאים הבאים:

תנאי האישור

1) הניסוי הרפואי יבוצע לפי העקרונות של הצהרת הלסינקי ועל-פי דרישות הנוהל של ניסויים רפואיים בבני אדם בישראל (2014) ודרישות הנהלים הבין-לאומיים העדכניים.

2) הטיפול יינתן רק לאחר מתן הסבר למטופל או לנציגו החוקי והחתמתו על טופס ההסכמה מדעת שצורף לבקשה.

3) כל שינוי, תוספת או חריגה מפרוטוקול הניסוי הרפואי, טעון אישור בכתב של ועדת הלסינקי של המוסד הרפואי ו/או של משרד הבריאות.

4) על החוקר הראשי בניסוי הרפואי לדווח לוועדת הלסינקי של המוסד הרפואי וליזם על כל אירוע חריג רציני (SAE) שארע במהלך הניסוי הרפואי (כמפורט בפרק 13 בנוהל), או על הפסקת הניסוי (כמפורט בפרק 15 בנוהל). ועדת הלסינקי המוסדית תבדוק את הדיווח ותעביר את חוות-דעתה למשרד הבריאות.

5) הארכת תוקף הניסוי הרפואי: **שלושה חודשים בטרם חלוף התקופה המאושרת לניסוי הרפואי**, חובה על החוקר הראשי להעביר דו"ח התקדמות על מהלך הניסוי לוועדת הלסינקי של המוסד הרפואי. הוועדה תודיע על החלטתה לגבי המשך הניסוי למנהל המוסד הרפואי. המנהל ינפיק אישור חדש לניסוי הרפואי.

6) בתום הניסוי הרפואי יגיש החוקר הראשי, לוועדת הלסינקי דו"ח מסכם על מהלך הניסוי ותוצאותיו.

7) האישור ניתן לחוקר הראשי ולמוסד הרפואי המצוינים לעיל ואינו ניתן להעברה לאחר.

8) בניסויים רפואיים הכרוכים במתן שירותים: ביצוע בדיקות רפואיות או באספקת אביזרים, תכשירים רפואיים או משתלים, חובה על החוקר הראשי להודיע לרופא המטפל בחולה בקהילה על השתתפותו בניסוי.

9) אין לפרסם כל מידע אודות הניסוי הרפואי באמצעי התקשורת ההמוניים, כגון עיתונות, רדיו, טלוויזיה, אינטרנט, למעט פרסום בעיתונות מדעית או בכנסים מדעיים, ולמעט פרסום לצורך גיוס המשתתפים בניסוי.

10) אספקת מוצר המחקר (Investigational Product – IP) או האמ"ר למוסד הרפואי בו נערך הניסוי הרפואי היא באחריות יזם הניסוי. אחסונו וניפוקו של מוצר המחקר למטופלים הם באחריות החוקר הראשי. במקרים של תכשירים רפואיים, פעולות אלו יבוצעו באמצעות בית המרקחת המוסדי, אלא אם כן ועדת הלסינקי החליטה אחרת.

11) שמירת מסמכים: יש לשמור את כל מסמכי הבקשה, האישורים וכל המסמכים הנאספים במהלך הניסוי הרפואי **לפחות 15 שנים מתום הניסוי**.

12) הגבלות נוספות:

**שם הנוהל: נוהל לניסויים רפואיים בבני-אדם**	**תאריך: מאי 2014**
**טופס 7**	
**אישור מנהל המוסד הרפואי לביצוע ניסוי רפואי**	

(13) תוקף האישור: 18/5/17

בהצלחה!

בכבוד רב,

ד"ר יעקב פולאקביץ
מנהל המוסד הרפואי

העתק: יו"ר ועדת הלסינקי
מנהל בית המרקחת
יזם הניסוי / נציגו בארץ (באמצעות החוקר)
המחלקה לניסויים קליניים, אגף הרוקחות-משרד הבריאות
