# Supplementary material for: An accurate wearable hydration sensor: Real-world evaluation of practical use
Source: PLoS One. 2022 Aug 24;17(8):e0272646. doi: 10.1371/journal.pone.0272646 (PMC9401113; doi:10.1371/journal.pone.0272646)
Supplement: S3 File — (PDF) [file pone.0272646.s005.pdf]

שם הנוהל: נוהל לניסויים רפואיים בבני-אדם	תאריך: מאי 2014
**טופס 6**	
**אישור ועדת הלסינקי לביצוע ניסוי רפואי**	

טירת כרמל, 03.05.17

לכבוד
פרופ' אנטולי קריינין, החוקר הראשי
מנהל מחלקה 5א'

הנדון: **אישור ועדת הלסינקי**

שוכנענו שהניסוי הרפואי, אשר פרטיו מופיעים להלן, אינו נוגד את עקרונות הצהרת הלסינקי, תקנות בריאות העם (ניסויים רפואיים בבני-אדם) תשמ"א-1980 ונוהל לניסויים רפואים בבני-אדם 2014.
אישור זה הנו שלב ביניים בהליך אישור הניסוי הרפואי. החוקר יוכל להתחיל בביצוע הניסוי רק לאחר קבלת אישור המנהל (טופס 7).

פרטי הניסוי

מספר בקשה בוועדה מוסדית: **04/17**	סוג הניסוי: **אמ"ר**.
נושא הניסוי : **אימות פונקציונלי של חיישנים ביולוגיים עבור מעקב פיזיולוגי.**	
שם מוצר המחקר: **Spectrophon Dehydration Body Monitor based on Samsung PPG/HRM Gear 2S**	שם היצרן: **Spectrophon Ltd.**
ניסוי רב-מרכזי בארץ: X **לא**	

מסמכי הניסוי

פרוטוקול הניסוי- שם/מספר:	גרסה: **3**	תאריך: **27.03.17**
טופס הסכמה- שם/מספר:	גרסה: **3**	תאריך: **31.03.17**
חוברת לחוקר- שם/מספר:	גרסה: **2**	תאריך: **02.04.17**
מסמך איכות מוצר - שם/מספר:	גרסה:	תאריך:
טופס 11- גרסה: **1**	תאריך: **18.04.16**	

שם הנוהל: נוהל לניסויים רפואיים בבני-אדם	תאריך: מאי 2014

טופס 6

אישור ועדת הלסינקי לביצוע ניסוי רפואי

הניסוי הרפואי הנו

X **ניסוי רפואי מיוחד, שבסמכות מנהל המוסד הרפואי לאשרו ללא אישור נוסף של משרד הבריאות.**

תנאים והגבלות: אין

חריגה מדרישות הנוהל, שאושרה: אין

שם יו"ר ועדת הלסינקי	חתימה	תאריך הדיון	תאריך האישור
**ד"ר רון פלד**		**25.04.17**	**03.05.17**

הערה: אישור זה מהווה תנאי לרישום באתר ה-NIH. החוקר יעביר את מס' הרישום לוועדת הלסינקי.

העתק: מנהל המוסד הרפואי
המחלקה לניסויים קליניים, אגף הרוקחות- משרד הבריאות
