# Supplementary material for: An accurate wearable hydration sensor: Real-world evaluation of practical use
Source: PLoS One. 2022 Aug 24;17(8):e0272646. doi: 10.1371/journal.pone.0272646 (PMC9401113; doi:10.1371/journal.pone.0272646)
Supplement: S4 File — (PDF) [file pone.0272646.s006.pdf]

Translation, IRB Document 1

**Name of the procedure: A procedure for experiments on humans**	**Date: May 2014**

Form 7

Approval of the director of the medical institution to perform a medical experiment

Date: 8 May 2017

In honor of
Prof. Anatoly Kreinin
Chief investigator
Head of Department 5aleph

Prof / Dr. distinguished/distinguishedess,

Subject: **Approval to perform a medical experiment on humans**

In accordance with your application dated: March 27, 2017, this approval was given for performing the medical experiment according to the application documents.

Details of the experiment

Request number at an institutional committee: **04/17**	Type of experiment: **MRS**
Application number at the Ministry of Health:	NIH Registration Number:
Subject of the experiment: **Functional verification of biological antecedents for physiological follow-up.**	
Research product name: **Spectrophon Dehydration Body Monitor based on Samsung PPG / HRM Gear 2S**	Manufacturer Name: **Spectrophon Ltd.**
Multi-central experiment in Israel: **X no**	

Experimental documents

Experiment protocol - name / number:	Version: **3**	Date: 27 Mar 2017
Consent form - name / number:	Version: **3**	Date: 31 Mar 2017
Booklet for the researcher - name / number:	Version: **2**	Date: 2 Apr 2017
Product Quality Document - name / number:	Version:	Date:
Form 11 - Version: 1		Date: 18 Apr 2016

By the power vested in me by the Director General of the Ministry of Health, as the “director” authorized to approve clinical trials in human beings, at the medical institution, after your application has been approved by the institutional Helsinki committee on: **03 May 2017** /after the application has been approved by the Ministry of Health and after having been convinced that the clinical trial complies with the principles of the Helsinki Declaration and the Public Health Regulations (Clinical Trials in Human Beings) 1980, and that the contract between the sponsor, the principal investigator and the medical institution complies with the requirements of the Procedure for Clinical Trials in Human Beings, I hereby approve to conduct the trial, subject to the following conditions:

Conditions of Approval

1) The clinical trial will be conducted in accordance with the principles of the Helsinki Declaration, the requirements of the Procedure for Clinical Trials in Human Beings in Israel (2014), and the requirements of current international procedures.
2) Treatment will only be administered after an explanation has been given to the patient or legal representative thereof, and after the patient or said representative have signed the informed consent form attached to the application.
3) Any amendment or addition to the clinical trial protocol or any deviation thereof will require written approval by the medical institution’s Helsinki committee and/or the Ministry of Health.
4) The clinical trial’s principal investigator is to report to the medical institution’s Helsinki committee and to the sponsor of any serious adverse event (SAE) that occurs during the clinical trial (as specified in article 13 of the procedure), or of trial discontinuation (as specified in article 15 of the procedure). The institutional Helsinki committee will review the report and forward its statement of opinion to the Ministry of Health.
5) Extending validity of the clinical trial: three months prior to the expiration of approval issued for the clinical trial, the principal investigator must forward the medical institution’s Helsinki committee a progress report describing the progression of the trial. The committee will make its decision regarding the continuation of the trial known to the director of the medical institution. The director shall issue a new approval for the clinical trial.
6) Upon completion of the clinical trial, the principal investigator will submit to the Helsinki Committee a summary report of trial progress and results.
7) The approval is issued to the aforementioned principal investigator and medical institution, and cannot be transferred to others.
8) In clinical trials involving provision of services: medical tests to be conducted or medical equipment, medical agents or implants to be supplied, the principal investigator is obliged to inform the attending physician in the community of the patient’s participation in the trial.
9) No information regarding the clinical trial shall be released to the mass media, e.g. the press, radio, TV and Internet, except publications in scientific magazines or medical conventions, and except publication required to recruit trial participants.

10) Supply of the investigational product (IP) or medical device to the medical institution where the clinical trial is conducted, is the responsibility of the trial sponsor. IP storage and dispensing to patients are the responsibility of the principal investigator. When medications are concerned, such actions will be performed in accordance with the institutional pharmacy, unless otherwise decided upon by the Helsinki committee.

11) Retention of documents: all application documents, approvals and all records collected during the course of the clinical trial must be kept for at least 15 years as of trial completion.

12) Other limitations:

13) Approval valid: 7/May/2018_

G o o d L u c k !

Sincerely,

Dr. Yakov Folakbitz

Director of the Medical Institution

CC: Helsinki Committee Chairman
Pharmacy Director
Trial Sponsor / its representative in Israel (through the investigator)
Clinical Trial Division, Pharmaceutical Administration - Ministry of Health
