# Supplementary material for: An accurate wearable hydration sensor: Real-world evaluation of practical use
Source: PLoS One. 2022 Aug 24;17(8):e0272646. doi: 10.1371/journal.pone.0272646 (PMC9401113; doi:10.1371/journal.pone.0272646)
Supplement: S5 File — (PDF) [file pone.0272646.s007.pdf]

**Procedure Name: Procedure for Human Medical Experiments**	**Date: May 2014**
**Form 6**	
**Approval of the Helsinki Committee for a Medical Experiment**	

Carmel Castle, 03.05.17

for
Prof. Anatoly Crane, Principal Investigator
Department Manager 5A

In **question:Approval of the Helsinki Committee**

We were convinced that the medical trial, the details of which are listed below, does not contravene the principles of the Helsinki Declaration, the People's Health Regulations (Human Health Experiments) 5740-1980, and a procedure for human clinical trials in 2014.
This approval is an intermediate stage in the medical trial approval process. The researcher will only be able to begin the experiment after receiving the approval of the manager (Form 7).

Details of the experiment

Number of applications in an institutionalcommittee: **04/17**	Type of experiment: **M.R.S.**
The subject of the experiment: **Functional validation of biological sensors for physiological surveillance.**	
Research Product Name**: Spectrophon Dehydration Body Monitor based on SamsungPPG/HRMGear 2S**	Manufacturer name: **Spectrophon Ltd.**
Multicenter experiment in Israel: **X No.**	

Test documents

Test-name/numberprotocol:	**Version:3**	**Date:27.03.17**
Consent Form-Name/Number:	**Version:3**	**Date:31.03.17**

Procedure Name: Procedure for Human Medical Experiments	Date: May 2014
**Form 6**	
**Approval of the Helsinki Committee for a Medical Experiment**	

Researcher- name/numberbrochure:	**Version:2**	**Date:02.04.17**
Product Quality Document - Name/Number:	Version:	Date:
Form 11- **Version:1**	**Date:18.04.16**	

The medical experiment is

X A special medical trial, which is the authority of the director of the medical institution to approve it without further approval from the Ministry of Health.

terms and conditions:none

Procedure requirements exceeded, approved:None

Name of Chairman of the Helsinki Committee	signature	Date of discussion	Approval date
**Dr. Ron Peled**	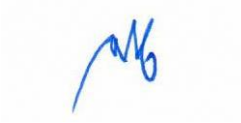	**25.04.17**	**03.05.17**

Procedure Name: Procedure for Human Medical Experiments	Date: May 2014
**Form 6**	
**Approval of the Helsinki Committee for a Medical Experiment**	

Note: This certificate is a condition for registration on theNIHwebsite. Theinvestigator will transfer the registration number to the Helsinki Committee.

Copy: Director of the Medical Institute
Department of Clinical Trials, Pharmacy Division - Ministry of Health
